# COVID-19 vaccine hesitancy among unvaccinated individuals in a primary care setting, Pretoria

**DOI:** 10.4102/safp.v66i1.5988

**Published:** 2024-09-23

**Authors:** Dikonketjo M.P. Moeti, Indiran Govender, Tombo Bongongo

**Affiliations:** 1 Department of Family Medicine and Primary Health Care, School of Medicine, Sefako Makgatho Health Sciences University, Pretoria, South Africa

**Keywords:** COVID-19 vaccine hesitancy, unvaccinated individuals, primary health care setting, vaccine safety concerns, Pretoria, South Africa

## Abstract

**Background:**

South Africa faced challenges while implementing coronavirus disease 2019 (COVID-19) measures such as mass vaccination. Some people rejected or were hesitant to receive government-recommended vaccines. This study explored COVID-19 vaccination hesitancy among unvaccinated individuals in a primary care setting in Pretoria, South Africa.

**Methods:**

This was an exploratory phenomenological study that included one-on-one interviews with 12 individuals at Temba Community Health Centre in Pretoria, South Africa.

**Results:**

The research revealed five themes: perceptions of COVID-19 disease, perceptions of COVID-19 vaccine, factors related to non-vaccination, information sources about the COVID-19 vaccine, and long-term vaccination decisions. There were seven linked sub-themes.

**Conclusion:**

Overall, participants had a good understanding of COVID-19 disease, but limited knowledge about the vaccine, causing hesitancy to get vaccinated. Reasons for not getting vaccinated included health-related concerns, safety concerns, personal experiences, and social and political factors. Safety and health-related concerns were prevalent, with adverse vaccine outcomes being the most common concern. Most participants had experienced a historic encounter with a vaccine-related death or illness.

**Contribution:**

Vaccine hesitancy should be viewed as a powerful concern from the community, and a key source of worry for the health authorities over any vaccine-related doubt.

## Introduction

The World Health Organization (WHO) declared the coronavirus disease 2019 (COVID-19) a pandemic in 2020,^1^ leading to South Africa’s lockdown in March 2020.^2,3^ Vaccination remains the most effective method to prevent mortality and improve community protection against severe acute respiratory syndrome coronavirus 2 (SARS-CoV-2) through herd immunity.^4^

According to the WHO, vaccine hesitancy is the delay or refusal to receive a vaccine, notwithstanding its availability and accessibility.^1^ Such behaviour was identified as one of the biggest concerns to public health in 2019.^5^ A worldwide survey discovered that individuals with unfavourable opinions about the COVID-19 vaccine included young people living in cities, respondents who do not know anyone who has tested positive for the virus, those who believe the risk is overstated, women, and conspiracy theorists.^6^ In a Malaysian study, respondents with higher levels of education, who were female, young, and free of chronic illnesses, had more readiness to obtain the vaccination.^7^ This is consistent with the results of an international study conducted in Africa.^6^

In South Africa, some studies reveal that vaccination hesitancy is a highly variable, complex, context-specific, social phenomenon and has been influenced by factors such as complacency, convenience, and confidence in getting vaccinated.^8,9,10^ The decision to get vaccinated has been demonstrated to be impacted by a variety of factors, including vaccine availability and accessibility, perceived health risk, perceived vaccine safety, and socio-demographic characteristics.^4^ Other studies in South Africa had contrasting findings with regard to the relationship between educational level and vaccine acceptance rates.^11,12^ Research has further indicated that vaccine acceptability is higher in urban as compared to rural areas.^10^

Understanding the reasons why certain individuals are willing to accept the COVID-19 vaccine without much hesitancy could help motivate those who are hesitant to vaccinate and help increase the likelihood of vaccine uptake in the country,^10^ thereby permitting ‘herd immunity’ to occur.^4^ As some studies have indicated, individual decisions to be vaccinated will be influenced by the perceived risks of vaccination and the perceived threat of disease.^8,9,10^ South Africans who are unwilling to be vaccinated express concerns about the vaccine’s side effects, the use of new deoxyribonucleic acid and/or messenger ribonucleic acid (DNA and/or mRNA) platforms, the lack of long-term safety records, the vaccine’s effectiveness against new variants of the virus, the duration of actual protection that a vaccine can provide, and the fact that the vaccine is moving through clinical trials too quickly.^10,11,12,13,14^ The most common motive for vaccination was the desire to protect oneself and one’s family members.^3,11^ The political and health authorities must devise plans to help communities combat the pandemic.^9,10^ Therefore, it would be beneficial to understand how the South African community views the COVID-19 vaccine, in order to design and implement risk communication and community engagement strategies.^12,15^ These strategies will guide interventions aimed at promoting and sustaining acceptance of COVID-19 vaccines while encouraging compliance with other COVID-19 preventive measures.^9,10^ Addressing community mistrust and promoting two-way communication between government and the communities it oversees are essential for a successful response to the COVID-19 pandemic.^9,10^

It is necessary to implement tailored methods that directly address the unique issues that differing socio-demographic groups face.^9,10^ To meet the requirements of specific populations like migrants or refugees, these resolutions could require extension.^6,9,10^ To completely comprehend the priorities and views of various demographic groups, further studies are necessary.^9^ Observing that many people in Temba, Pretoria are not vaccinated despite the fact that COVID-19 vaccination services were readily available and accessible in this community inspired the current study, which explored vaccination hesitancy among unvaccinated individuals attending a primary healthcare facility in Pretoria.

## Research methods and design

### Study design

This was an exploratory phenomenological study that involved one-on-one interviews with 12 respondents from Temba Community Health Centre in Pretoria, South Africa.

### Study setting

The study was conducted at Temba Community Health Centre in Pretoria community health centre (CHC) in Hammanskraal, Pretoria. The neighbourhood is in the northern part of Pretoria, around 60 km from the Pretoria City Centre, on the N1 national road. This CHC is one of two COVID-19 vaccination facilities in the vicinity. The CHC is open 24 h a day and provides a wide range of health services. The clinic employs a family physician, general practitioners or medical officers, medical interns, first-year family medicine registrars, professional nurses, and auxiliary nurses.

### Study population, sampling technique and sample size

This study included all Temba Community Health Centre in Pretoria CHC patients aged 18 years and above who were eligible for the COVID-19 vaccine but had not yet received it. A retired nurse was trained as a research assistant (RA) by the principal author; this regarding on how to present the study, its aim and objectives to patients while they were waiting for their files in the waiting area of the CHC. This took the entire month of March 2024. The sample size was established using a purposive sampling strategy based on unvaccinated persons’ desire to engage in the research as well as data saturation (when the same themes, ideas, or patterns appear).^15^ Applying the inclusion criteria, 12 participants were selected for this study (*N* = 12).

### Data collection

The RA conducted all the interviews using the interview guide. This interview guide was piloted at Phedisong 4 CHC in Ga-Rankuwa, approximately 60 km from Temba Community Health Centre in Pretoria CHC. Phedisong 4 CHC was selected because it is a CHC like Temba Community Health Centre in Pretoria, provides the same services, was a COVID-19 vaccine centre, and users share the same culture (language, food habits, etc.) as Temba Community Health Centre in Pretoria patients. The findings of this pilot research helped the principal author, and co-authors adjust the interview guide as needed and better grasp the study’s aims. The interview guide was developed by the principal author in English and Tswana, the two languages spoken in the study area. The RA, who is fluent in both languages, assisted with the back translation. Dates and times were scheduled for each participant’s one-on-one interview, which lasted 40 min – 50 min, particularly for those who could not wait to be interviewed the same day. Interviews were conducted at Temba CHC following a schedule developed together by the RA, the principal author, and each participant. Some participants were interviewed on the same day they were chosen, while others were given a date and time that was convenient for them. Before the interview, each participant signed an informed consent form and supplied information about their age, sex, occupational status, marital status, employment status, level of education, religion, number of children, and chronic health issues. The interviews were audio recorded and started with open-ended questions, followed by prompted questions. Conversations in Tswana were translated into English at the end of the interview by the RA and principal author who participated in the interviews, and both were fluent in both languages.

### Data analysis

The participants’ socio-demographic characteristics were recorded and coded in a Microsoft Excel spreadsheet before being exported to Stata 14, where descriptive analysis was conducted. A frequency table was utilised to present the participants’ socio-demographic information.

The audio recordings from the interviews were transcribed into written text using Microsoft Word, then edited and confirmed for accuracy. The cleaned transcripts were then exported onto NVivo 12 for qualitative analysis. Using interpretative philosophy, the verbatim responses provided by participants were identified and coded. The first few transcripts yielded initial codes, which were then utilised to combine related concepts or responses. A code book was used to specify the codes, which were then merged as needed to build themes and sub-themes. Five themes were discovered. The socio-demographic characteristics of the participants attempted to address the first objective, which was to describe socio-demographic variables. The first, second, and third themes address the objectives of exploring participants’ comprehension of COVID-19 disease and the vaccine. The fourth theme centred on the objective of investigating sources of information on the COVID-19 vaccine, while the final theme discussed long-term choices regarding the COVID-19 vaccine.

### Ethical considerations

Ethical clearance to conduct this study was obtained from the Sefako Makgatho Health Sciences University, Research Ethics Committee (No. SMUREC/M/22/2022:IR). Additionally, permission was acquired from the Temba CHC’s Operational Manager. To protect patient confidentiality, each participant was given a code; their real names were not used in the research.

## Results

### Socio-demographic variables

The total sample included 12 participants, with 10 females and two males. The mean age was 32.2 years, with the youngest participant being 20 years and oldest 55 years. Most of the participants were single (*n* = 10; 83.33%), unemployed (*n* = 7; 58.33%), and had completed matric (*n* = 5; 41.67%). Moreover, half of the participants had only one child (*n* = 6; 50.00%) and almost all (*n* = 11; 91.67%) identified with the Christian religion. A third (*n* = 4; 33.33%) of the participants had a chronic condition (see Table 1).

**TABLE 1:** Socio-demographic variables of participants.

Variables	Frequency (*n*)	Percentage (%)	Mean	s.d.	Min	Max
**Age (years)**			**32.2**	**10.32**	**20**	**55**
20–24	4	33.33	-	-	-	-
25–29	3	25.00	-	-	-	-
≥ 30	5	41.67	-	-	-	-
**Sex**						
Male	2	16.67	-	-	-	-
Female	10	83.33	-	-	-	-
**Marital status**						
Married	2	16.67	-	-	-	-
Single	10	83.33	-	-	-	-
**Number of children**						
None	2	16.67	-	-	-	-
One	6	50.00	-	-	-	-
≥ 2	4	33.33	-	-	-	-
**Highest level of education**						
Primary school	1	8.33	-	-	-	-
High school	3	25.00	-	-	-	-
Completed matric	5	41.67	-	-	-	-
Tertiary	2	16.66	-	-	-	-
Postgraduate	1	8.33	-	-	-	-
**Employment status**						
Employed	3	25.00	-	-	-	-
Unemployed	7	58.33	-	-	-	-
Student	2	16.67	-	-	-	-
**Religious affiliation**						
Non-Christian	1	8.33	-	-	-	-
Christianity	11	91.67	-	-	-	-
**Chronic illness**						
Yes	4	33.33	-	-	-	-
No	8	66.67	-	-	-	-

s.d., standard deviation; Min, minimum; Max, maximum.

### Themes and sub-themes

Five themes were identified: perceptions of COVID-19 disease, perceptions of COVID-19 vaccine, factors associated with non-vaccination, information sources about the COVID-19 vaccine, and long-term vaccination decisions. Seven related sub-themes emerged from these themes (see Table 2).

**TABLE 2:** Themes and sub-themes.

Themes		Sub-themes
1.	Perceptions of COVID-19 disease	1.1. Understanding of COVID-19 disease
2.	Perceptions of COVID-19 vaccine	2.1. Understanding of COVID-19 vaccine 2.2. Views about the benefits of COVID-19 vaccine
3.	Factors related to non-vaccination	3.1. Personal experiences 3.2. Health-related factors 3.3. Societal and political factors 3.4. Safety concerns
4.	Information sources about the COVID-19 vaccine	-
5.	Long-term decisions regarding vaccination	-

COVID-19, coronavirus disease 2019.

### Theme 1: Perceptions of COVID-19 disease

#### Sub-theme 1: Understanding of COVID-19 disease

Most of the participants had adequate knowledge of COVID-19 disease and were well-versed in some of the symptoms experienced. To illustrate, one participant stated:

‘It’s like a dangerous flu. A very dangerous flu, you know? Yes, a dangerous flu, that if you get it, you know what? It’s going to kill you. If you happen to get that flu, you’d have to make sure that you get it treated quickly.’ (24 years old, female, high school, unemployed)

This statement was supported by another participant, who added:

‘According to me, I see it as a disease, according to the way they explained, a very dangerous disease. COVID-19 is like flu. The symptoms are like headaches, sweating at night, coughing, yeah, so you must go to the clinic and get tested because it’s dangerous.’ (42 years old, female, high school, unemployed)

There were others, however, who held controversial views about the virus, indicating that it was man-made and was an attempt to minimise the population:

‘I feel like it’s a man-made disease.’ (24 years old, female, tertiary, employed).

This statement was supported by another participant, who stated:

‘It was man-made. I never believe that COVID was real, and I won’t change my opinion tomorrow even if they want to arrest me. It’s fine, I can do the time in jail for their COVID.’ (29 years old, female, matric, student)

When expressing how he first heard about the virus, one participant reported:

‘I don’t know because the first time we heard of COVID is, the theory that I heard was some billionaire, I can’t remember his name, created this virus to, you know, especially people in Africa, to kind of minimise; and in China, because we know the population in China is pretty big, you know, to minimise the population.’ (27 years old, male, postgraduate, unemployed)

However, unlike the preceding female participants, he did not believe this theory and said, ‘I personally thought it was very bogus, didn’t really believe it’ (27 years old, male, postgraduate, unemployed). When asked if the virus is still there, he replied as follows:

‘I really don’t know right now, because I don’t know what you think by all that. Do I think it’s still there? I think there’s a possibility of it resurfacing, because it is a virus. I don’t think viruses just disappear out of thin air. So, I do think there’s a possibility one day we would hear that there is a serious case of it again.’ (27 years old, male, postgraduate, unemployed)

The same sentiments were shared by a 39-year-old participant, who responded:

‘I also ask myself all the time, because according to my knowledge, it hasn’t stopped. COVID hasn’t stopped. It doesn’t mean just because it’s quiet that COVID-19 isn’t here anymore totally, as some people will claim. It hasn’t ended. But I can see that there are no hard measures that have been taken to prevent the infection from coming again.’ (39 years old, female, matric, unemployed)

Overall, most participants had a clear understanding of COVID-19 disease, with only two participants who were doubtful about its existence.

### Theme 2: Perceptions of COVID-19 vaccine

#### Sub-theme 2.1: Understanding of COVID-19 vaccine

Along with awareness of COVID-19 disease, participants also had a rudimentary comprehension of the COVID-19 vaccine. It was shown that regardless of educational attainment, most participants could describe the vaccine in terms of their own understanding. The following summarises some of the responses to the question of what the COVID-19 vaccine is:

‘Yes, a vaccine is what was used to treat those who had COVID. They said it heals. And we all must get injected so that we shouldn’t get COVID.’ (22 years old, female, high school, unemployed)

‘The purpose of getting vaccinated, in my own understanding, was to prevent those who hadn’t been infected from getting infected. So maybe because people got infected even after they got vaccinated, I don’t know where or what went wrong. But their purpose was that those that hadn’t gotten vaccinated shouldn’t get it; it was a prevention from getting COVID-19.’ (24 years old, female, tertiary, employed)

‘Isn’t they said it protects against COVID?’ (55 years old, female, matric, unemployed)

Others seemed unsure when first asked; for example, one female replied, ‘I can’t explain, I can’t explain’ (24 years old, female, high school, unemployed). However, when probed further about her understanding, she responded as follows:

‘So they explained that they vaccinate you so that if you are infected from someone, it doesn’t really infect you like that, like it doesn’t come on as strong. But otherwise, it won’t do anything. But for those who aren’t vaccinated, when it comes, it comes all at once and very strong. And if you don’t get help quickly or come to the clinic, it’ll kill you.’ (24 years old, female, high school, unemployed)

Half of the participants were familiar with other types of vaccines, mostly those administered during childhood, whereas two indicated that this was the first time they ever heard about a vaccine. It was discovered that although participants had a basic understanding of the vaccine, there was a lack of understanding of what exactly happens in the body when the COVID-19 vaccine is administered or what the vaccine is made of. To illustrate this, a 55-year-old female participant answered:

‘I don’t know, they didn’t explain to me what the vaccine does. But according to my understanding, they explained that you are getting vaccinated against COVID. So that you can protect yourself, so that it doesn’t kill you.’ (55 years old, female, matric, unemployed)

When she was asked to explain what it does in the body, she responded by saying, ‘It goes with the blood so that you don’t have COVID or anything.’

#### Sub-theme 2.2: Views about the benefits of COVID-19 vaccine

Divergent opinions existed on the advantages of the vaccine: while some believed it to be helpful in preventing COVID-19, others questioned its safety and effectiveness. For example, one participant said:

‘I feel as if the money that was used could have been used to do more research about COVID-19 so that we can see exactly what permanent solution we can come up with to give people to be completely cured from COVID-19. Not this prevention approach that we’ve got from the vaccination, we need something that’s going to work.’ (24 years old, female, tertiary, employed)

In addition, when asked if there is a difference between people who are vaccinated and those who are not, she responded:

‘No according to me, I didn’t see any benefits because I didn’t get vaccinated and I’m still here. There were those who got it and are no longer with us. So, for me, it’s all the same.’ (24 years old, female, tertiary, unemployed)

One participant gave a balanced view, and said:

‘Yoh, that’s a tough one. That’s a tough one. I honestly don’t know how to answer that. But I think I would answer for the unvaccinated. I think that I’m better off because of my allergies and sinuses. But for somebody else, I’m coming back to support the one who got vaccinated. For instance, for someone who has respiratory problems like asthma and such. When they get a cold, they get easily blocked, so if they are vaccinated and they don’t experience any problems. I believe that you are covered and safe once you get it. So, I don’t only look at it from my own point of view. I also look at it from another person’s point of view in terms of how it benefits that person.’ (38 years old, female, tertiary, employed)

For the patients with chronic conditions, one participant commented as follows:

‘I’d recommend that they get it, because honestly it will help them so that they can get stronger. They shouldn’t get infected by all these illnesses whereby someone is in and out of the hospital. As well as the case in COVID-19, they were in and out of hospital, even here at the clinic we had regular cases that needed transfer. It’s not nice.’ (38 years old, female, tertiary, employed)

This view came from a 38-year-old professional nurse, and her background may have aided in her arriving at this balanced view.

### Theme 3: Factors related to non-vaccination

Four major factors influenced individuals’ decisions not to be vaccinated: health-related factors, safety concerns, personal experience, and a variety of social and political variables. Most participants raised health and safety concerns. Those with health issues had chronic illnesses and were frightened of negative health outcomes because of their compromised immunity. Participants had worries about the efficacy and safety of the vaccine. Negative vaccine outcomes, such as illness or death, were a major concern, even if family vaccination status did not significantly impact vaccination decisions. Most of the individuals made decisions based on their fear of tragedies that had happened to others also happening to them.

#### Sub-theme 3.1: Personal experiences

*Inadequate knowledge of vaccine contents*: Despite having sufficient understanding about the vaccine, most participants acknowledged that they were hesitant to get vaccinated as they did not know what the vaccine was made of. In response to the question ‘What is it made of?’, one participant stated:

‘No, I don’t know. That’s why I told you that I asked myself once what it’s actually made of, because I can see the effect it has on my mother-in-law, that it’s making her sick. So, what is it going to do when it enters my body?’ (38 years old, female, matric, unemployed)

A similar response was noted from another peer, who replied:

‘We don’t know what it is, and we don’t know where it comes from. So just imagine me taking something and putting it in my body, and I don’t even know what it is.’ (39 years old, female, matric, unemployed).

Another participant stated as follows:

‘That’s why we don’t have a lot of information, to be honest. We just hear things on the radio, clinic, stadium. When you get there, they won’t tell you what this thing is made of. They’ll just inject you and give you a paper to sign and tell you to come back.’ (42 years old, female, high school, unemployed)

*Disbelief/doubting the existence of COVID-19*: Disbelievers in COVID-19’s existence are more likely to avoid vaccination, because of distrust of the vaccine’s benefits, safety, and efficacy, leading to a lack of vaccination. One participant said:

‘Okay, for me on why our people don’t want to get vaccinated, I’ll relate mostly on the youth because I fall under that. Okay I didn’t vaccinate totally, totally. Because on my side I don’t believe that there was anything like COVID. And I never had to believe that COVID was ever there. It’s man-made.’ (29 years old, female, matric, student)

Additionally, the quick onset of the virus further spread doubt in the minds of the participants and the above participant further added:

‘That’s why I had thought it’s a man-made thing, because it came out of nowhere. Next thing already we have this COVID-19 virus, and we don’t know where it’s from or how it started. We just found ourselves getting sick out of nowhere, people like sick and dying. Where is it from? How? What happened?’ (29 years old, female, matric, student)

Some participants argued that these views were conspiracy theories, claiming that the coronavirus was real. A 27-year-old said:

‘I personally thought it was very bogus, didn’t really believe it did. Do I think COVID was really there? Yes, I did. Do I think it was a serious issue? Yes, I did. Did I think people, especially South Africans and old people around this area took it as serious as they were supposed to? No, they didn’t.’ (27 years old, male, postgraduate, unemployed)

Despite efforts by the government to encourage the public to vaccinate, by publicly broadcasting the vaccination of some political authorities, these were not well-received. As one participant complained:

‘… [*A*]nd then by the time they got the vaccine they didn’t vaccinate them in such a way that we as the community were able to … It was done on television. How could we be sure that that’s the same vaccine that they are giving to us? How did we know; even if I get vaccinated now and I become okay, is it the same as them? Can it be proven that what I was injected with is the same that was used for the political authorities?’ (29 years old, female, matric, student)

In addition, there were others who believed that the coronavirus was real, but maintained that it was not necessary to vaccinate because it was gone: ‘That disease isn’t here anymore, right? Yes! That disease is gone! Totally!’ (22 years old, female, high school, unemployed). Hence, they did not see the need to get the vaccine.

*Personal circumstances*: Some participants were just hesitant to take the vaccine, but were not necessarily anti-vaccine. They indicated that they were either not ready, were lazy, or wanted to determine if it was necessary: ‘But I never really; I wasn’t ready to go and get vaccinated’ (42 years old, female, high school, unemployed). Another indicated, ‘Yes, let me see if COVID is really going away or if it takes us or what, then I’ll come back’ (20 years old, female, matric, student).

Attributing the decision not to vaccinate being lazy, a 27-year-old participant said:

‘Okay. My biggest reason was, to be honest, procrastination. I procrastinate a lot sometimes. So, I had been meaning to go. And every single time I’m like, I wanna go. I wanna go. But I literally never went.’ (27 years old, male, postgraduate, unemployed)

The fear of the actual vaccination procedure also contributed as a participant admitted:

‘And another reason would be, a little bit afraid of needles, I’m afraid of needles. And I think, this for me as well at the same time; going to like a health facility or clinic, I went as a kid and then I was admitted and then I had an operation. So, I really don’t like health facilities, because as a kid I literally went for stomach cramps, and all of a sudden, they’re opening me up and stitching me the next day. So yeah, I really was not really, I’m not really a big fan of going to the clinic, even if it’s like for the littlest thing.’ (20 years old, female, matric, student)

#### Sub-theme 3.2: Health-related factors

The lack of vaccination among participants was primarily because of other medical conditions and a fear of getting infected by COVID-19. Chronic conditions like human immunodeficiency virus (HIV), diabetes, hypertension, and cancer, along with concerns about adverse outcomes because of allergies, contributed to this reluctance. Some illustrative comments were as follows:

‘If you have HIV, it’ll take you. So that’s why I’m so scared. Yoh, I won’t get vaccinated.’ (24 years old, female, high school, unemployed)

‘We are both chronic. So, I wasn’t going to be able to get vaccinated.’ (39 years old, female, matric, unemployed)

‘I got so sick that I ended up in hospital. But when they checked me, they found that I had diabetes. I think – I thought that it would make me sick as I saw how those who got vaccinated ended up sick. So, I thought to myself that because I have the same conditions if I go and get vaccinated …’ (55 years old, female, matric, unemployed)

‘I’m from a family with a lot of allergies. Yeah like, I recently stopped using penicillin because I got a rash. It’s so problematic. There are certain foods that I no longer eat. So, when I first heard about the vaccines, I thought to myself, if I go in there and get vaccinated, I’ll probably die. That’s the first thing that I thought of.’ (38 years old, female, tertiary, employed)

‘No, I don’t I’ve never seen anyone who has been sick from COVID-19. I’ve seen people who have posted on social media and were in hospital. And were in ICU. But to see someone who has COVID-19, no I haven’t. Even among my family members, no one has ever been infected with COVID-19.’ (24 years old, female, tertiary, employed)

Thus, not getting sick as well as not being exposed to immediate family or someone who had COVID-19 played a major role in not getting vaccinated, as participants did not see the urgency to do so. One participant even commented that she hardly followed the rules and regulations and still did not get infected:

‘We didn’t really obey the COVID rules. And most people I know are okay. I remember I used to travel from Themba to Rustenburg and there wasn’t even a problem. The taxi would be full to capacity and there was no problem, I was okay with it. I don’t remember even getting infected by COVID once.’ (29 years old, female, matric, student)

#### Sub-theme 3.3: Societal and political factors

Participants’ social environment significantly influenced their non-vaccination decision, with external influences like rumours, fake news, and misinformation causing negative views and opinions. For example, a 20-year-old student explained why she did not vaccinate despite testing positive for COVID-19:

‘Fear from people’s opinions. Because those who are watching news would be like, Oh, did you hear that after three years, people who get vaccinated are gonna die and stuff? And because of some of the effects of the vaccine you weren’t gonna realise them after three years.’ (20 years old, female, matric, student)

Another participant responded as follows regarding rumours:

‘Because I would hear rumours that this injection that people get kills them. If you find that you have … if your immune system is weak or have certain diseases in your body, you can die. That was the word on the street.’ (42 years old, female, high school, unemployed)

This participant further added:

‘You would hear different people talking, saying that the vaccine is killing people. And so, we lock ourselves in the house because we also don’t want to get sick.’ (42 years old, female, high school, unemployed)

However, it did seem as if some participants were aware of the negative impact of solely relying on other people for information. One participant indicated as follows:

‘Like when you hear people talking without full information, that thing kills you. So, you’d constantly be thinking that this is the reason why so-and-so passed away, that’s what was going on in our minds. So, you won’t go when you see people dying. But I didn’t really have a problem with it. I just kept on telling myself that one day I will go.’ (42 years old, female, high school, unemployed)

Word of mouth had far-reaching effects, because participants did not even need to have seen examples personally; as one male participant admitted, having never been exposed to someone who got vaccinated and had adverse effects: ‘No, I heard from other people’ (28 years old, male, primary, unemployed). Therefore, the spread of false information is potentially damaging, as in certain instances social opinions can override facts provided by authoritative sources. For example, a 24-year-old female indicated that despite the information provided by health professionals at health facilities, she decided not to get vaccinated because of other people’s opinions:

‘They would tell us, but we wouldn’t listen to them. We would only agree for that moment and then decide not to go when we’re alone. Yes, we heard from people and that made us scared.’ (24 years old, female, high school, unemployed)

The emergence of the COVID-19 pandemic amid the complex political climate in the country contributed significantly towards the decision not to vaccinate. Criticism of the governmental response to the COVID-19 pandemic was noted, as some participants expressed their discontent at being coerced to receive the vaccine. For example, one health professional noted:

‘Yes, I am a professional. We do get vaccinated for the likes of hepatitis and such, we get all types of vaccines and everything. But it was more like we were being forced, but I felt like, nope, I don’t want to.’ (38 years old, female, tertiary, employed)

Another participant commented as follows:

‘Actually, it’s like the government has this thing of wanting to minimise the population. It’s failing to maintain the country because everything goes into their pockets. So, they create diseases.’ (29 years old, female, matric, student)

#### Sub-theme 3.4: Safety concerns

Participants outlined various doubts and fears about the vaccine. For example, a male participant said, ‘Well, most of us black people didn’t trust what would happen if we got vaccinated’ (28 years old, male, primary, unemployed). Another participant said, ‘I’m scared of it! I’m scared of it’ (24 years old, female, high school, unemployed). Although she indicated that she did want to get vaccinated, her doubt overrode the desire: ‘I just had my doubts, but I did want it, you know? I wanted to, but I had doubts’ (24 years old, female, high school, unemployed).

Other participants were more focused on the safety of the vaccine itself, citing the fear of adverse effects it may have on them: ‘I wanted to see first what kind of side-effects people would get from getting vaccinated’ (24 years old, female, tertiary, employed). Some of the participants’ doubts were informed by knowing or hearing about someone who got sick or died from the vaccine. Almost all of the participants had a relative or loved one who got sick or died after receiving the vaccine:

‘A lot of people who have gotten vaccinated, for example, my brother, and he complained about a headache. So that’s why I’m scared of it. He was fine. but after getting vaccinated, that’s when they started getting complications. So that’s when I thought to myself, no, not going to happen.’ (39 years old, female, matric, unemployed)

Some mentioned that uncertainties about the vaccine’s safety were exacerbated by its accelerated release: ‘We are not even certain that those vaccines went through the proper channels or completed all of the stages necessary for a vaccine before being administered to humans, *due to* the time it took to procure them’ (38 years old, female, professional nurse).

### Theme 4: Information sources about the COVID-19 vaccine

Participants reported obtaining information from various sources, such as traditional, social, authoritative, and word-of-mouth sources, as reported by a 20-year-old female who shared her primary source of vaccine information: ‘TV, cell phones, social media’ (20 years old, female, matric, student). Likewise, another said: ‘We saw it on TV, on the news when they said something will come that will heal COVID’ (22 years old, female, high school, unemployed).

According to a report^9,10^ younger participants tend to rely more on social media as an information source:

‘Oh, honestly, my biggest source was the Internet. Like as a young person a lot was social media. Maybe clips that I would watch on YouTube or maybe the news sometimes, but a lot of information you get from social media.’ (27 years old, male, postgraduate, unemployed)

‘We just hear things on the radio, clinic, stadium’, said another participant (42 years old, female, high school, unemployed). Word of mouth was also used: ‘My parents and my siblings, so basically my family’ (20 years old, female, matric, student); ‘From the street, I’d hear it from people’ (24 years old, female, tertiary, employed). Others got informed by the healthcare professionals:

‘They would tell us, but we wouldn’t listen to them. We would only agree for that moment and then decide not to go when we’re alone.’ (24 years old, female, high school, unemployed)

### Theme 5: Long-term decisions regarding vaccination

The interviews revealed three main responses regarding the COVID-19 vaccine: those who would accept it, those who would not, and those who were still unsure. There was a similar number of participants who agreed that they would get vaccinated and those who said that they would not, except for two participants who indicated hesitancy. The following are responses from the first group: ‘Immediately’ (27 years old, male, postgraduate, unemployed); ‘Yes, I would agree to get vaccinated’ (28 years old, male, primary, unemployed). Other participants maintained their decision not to get vaccinated: ‘I’m still standing with my no’ (20 years old, female, matric, student); ‘I don’t think it’s the best option to get vaccinated’ (39 years old, female, matric, unemployed).

The two remaining participants showed hesitance, as one initially said no but changed her mind, stating:

‘I just had my doubts, but I did want it, you know? I wanted to but I had doubts. But to be honest, I’m not telling the truth. If it comes back, I’ll get vaccinated.’ (24 years old, female, tertiary, employed)

When asked what she would do if vaccination was made mandatory, one participant said, ‘Then I’m not sure then. I’m not sure if I’ll get vaccinated’ (22 years old, female, high school, unemployed). There were mixed feelings about getting vaccinated, as there was a balance between those who would go and those who refused.

## Discussion

The findings in this study mirrored those described in a worldwide survey that discovered that individuals with unfavourable opinions of the COVID-19 vaccine included young people living in cities, respondents who do not know anyone who has tested positive for the virus, those who believe the risk is overstated, women, and conspiracy theorists.^6^

Previous studies conducted in Asia and international studies in Africa found that the respondents with higher levels of education, who are female, young, and free of chronic illnesses, demonstrated more hesitancy to obtain the vaccination,^4,6,7^ mainly stating doubts about the efficacy of the vaccine, societal and political factors, and safety issues as their primary concerns. The same participants gave negative answers when asked if they would be willing to accept vaccination in the future, should the need arise.

In contrast, in this study, older, unemployed, female participants with lower levels of education were more predisposed than their younger counterparts to accept vaccination in future, should the safety and benefit of the vaccine be ascertained.

Most of the participants in this study had adequate knowledge of COVID-19 disease, and were well-versed in some of the symptoms experienced in those with COVID-19 infection, expressing an understanding of the reason for the lockdown in South Africa in March 2020,^2,3^ after the WHO declared COVID-19 a pandemic in 2020.^1^ Despite the public campaigns, information distribution and lockdown of 2020 in South Africa,^2,3,9,10^ it was discovered that participants in this study had no basic understanding of the COVID-19 vaccine. Although half of the participants were familiar with other types of vaccines, mostly those administered during childhood, two indicated that this was the first time they had ever heard about a vaccine.

Studies and information distributed by the government regarding the COVID-19 vaccine structure and origin, using new DNA and/or mRNA platforms,^2,3,10^ did not eliminate the lack of understanding of the participants regarding what exactly happens in the body when the COVID-19 vaccine is administered, or exactly what constitutes the COVID-19 vaccine. Conspiracy theories – as previously shown in other studies^2,3,8,9,10^ – greatly influenced COVID-19 hesitancy in this study group. Other participants held controversial views about the virus, saying that it was man-made and that it was an attempt to minimise the population, and two participants completely doubted the existence of the COVID-19 virus.

Other researchers^9,10^ have demonstrated that the social environment significantly influences participants’ decision not to vaccinate, citing external influences such as rumours, fake news, and misinformation as the source of their negative views and opinions regarding the COVID-19 vaccine.^9,10^ False information has been shown by research to be potentially damaging, as in certain instances, social opinions can override facts provided by authoritative sources.^2,3,8,9,10,15^ In this study, for example, a 24-year-old female indicated that despite the information provided by health professionals at health facilities, she decided not to get vaccinated because of other people’s opinions.

The governmental response to the COVID-19 pandemic has been criticised, with some participants feeling coerced into receiving the vaccine.^9,10^ This supports research studies that show that emergence of the COVID-19 pandemic occurred amid a complex political climate in South Africa, which significantly contributed towards the decision for non-vaccination.^2,3,8,9,10^ This finding also clearly illustrates and supports studies that demonstrated that, in South Africa, vaccination hesitancy is a highly variable, complex, context-specific, social phenomenon and that the decision to get vaccinated is impacted by a variety of factors, including vaccine availability and accessibility, perceived health risk, perceived vaccine safety, and socio-demographic characteristics.^2,3,8,9,10^

### Recommendation

A health authority-organised educational campaign that uses official channels to emphasise all pertinent vaccine information may raise public awareness and address vaccine hesitancy.

### Limitations

The results of this study cannot be generalised to a larger population because of its qualitative nature and the fact that it was conducted in a single community health centre in Pretoria.

## Conclusion

Participants had a good understanding of COVID-19 disease but had limited knowledge about the vaccine, which led to their hesitancy to vaccinate. Reasons for not getting vaccinated included health-related concerns, safety concerns, personal experiences, and social and political factors. Safety and health-related concerns were the most prevalent, with adverse vaccine outcomes being the most common. Most participants had a historical encounter with a vaccine-related death or illness, leading to them having a fear of similar outcomes and doubts about the vaccine’s efficacy.
